# Unveiling the Compression Mechanical Properties of AMPS–APTAC–DMAAm Terpolymeric Hydrogels

**DOI:** 10.3390/gels11120941

**Published:** 2025-11-24

**Authors:** Madina Mussalimova, Nargiz Gizatullina, Gaukhargul Yelemessova, Anel Taubatyrova, Gulzat Aitkaliyeva, Zhanserik Shynykul, Esra Su, Gaukhar Toleutay

**Affiliations:** 1 Department of Chemical and Biochemical Engineering, Geology and Oil-Gas Business Institute Named After K. Turyssov, Satbayev University, Almaty 050043, Kazakhstan; mussalimova.m@stud.satbayev.university (M.M.); gizatullinanargiz.nz@gmail.com (N.G.); gauhargul1997@gmail.com (G.Y.); 0ani.lani9@gmail.com (A.T.); g.aitkaliyeva@satbayev.university (G.A.); 2 Research Institute of Advanced Materials, Almaty 040000, Kazakhstan; 3 Department of Aquatic Biotechnology, Faculty of Aquatic Sciences, Istanbul University, 34134 Istanbul, Turkey; 4 Department of Chemistry, University of Tennessee, Knoxville, TN 37996, USA

**Keywords:** polyampholyte hydrogels, compression, swelling behavior, hydrogen bonding, AMPS, APTAC, DMAAm, crosslinking density, mechanical properties, Young’s modulus

## Abstract

Polyampholyte hydrogels are promising for load-bearing biomedical applications, but the link between composition and compression behavior remains unclear. In this study, we investigate how initial monomer concentration and a neutral comonomer influence swelling and mechanical properties in AMPS–APTAC networks. Terpolymeric AMPS–APTAC–DMAAm hydrogels were prepared with monomer concentrations from 1 to 2 M, MBAAm levels from 1 to 5 mol%, and DMAAm fractions from 0 to 0.16. Swelling was measured in water. Unconfined compression tests at 3 mm·min^−1^ provided stress–strain curves, Young’s modulus (E), fracture stress (σ_f_), fracture strain (ε_f_), and toughness (W) up to 99% strain. Increasing the monomer concentration produced denser networks, lower swelling, and higher stiffness. For C2M1, E reached 35.4 kPa, σ_f_ reached 0.8 MPa, ε_f_ was 82%, and W was 65.6 kJ·m^−3^. Adding DMAAm strengthened the gels through reversible associative interactions. At z = 0.06, σ_f_ increased to 4.28 MPa and W to 196.0 kJ·m^−3^. At z = 0.16, E increased to 103.0 kPa, while σ_f_ was 2.34 MPa and W was 191.6 kJ·m^−3^. Swelling decreased when monomer or crosslinker content increased. These results show that monomer concentration and DMAAm-mediated associations act as separate design variables that can be tuned to optimize stiffness, strength, and toughness in AMPS–APTAC polyampholyte hydrogels.

## 1. Introduction

**Figure 1:**
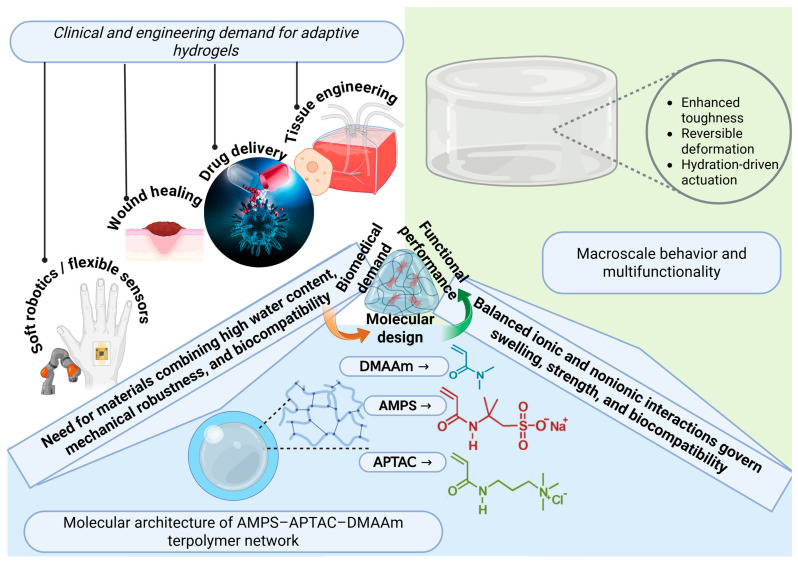
A schematic representation of the design rationale and biomedical relevance of adaptive polyampholyte hydrogels.

Hydrogels are three-dimensional crosslinked polymer networks with high water content and tissue-like environments. They are used in tissue engineering, drug delivery, biosensing, and soft robotic devices [1,2,3,4]. Their macroscopic behavior depends on monomer choice, polymer content, crosslink density, and associative interactions [1,2,3,4]. These design variables control swelling, stiffness, and energy dissipation and can be tuned for biomedical use [1,2,3,4] (Figure 1).

Polyampholyte and zwitterionic hydrogels contain oppositely charged or charge-neutral repeat units and show strong hydration, low fouling, and tunable mechanics [5,6,7,8,9,10,11,12,13,14,15]. Charge balance, ionic strength, and crosslink density govern volume changes, diffusion, and stiffness in these systems [10,11,12,13,14,15]. Reviews on zwitterionic and polyampholyte networks highlight robust hydration shells, reduced protein adsorption, and improved lubrication and device longevity [16,17,18,19,20]. Double-network and reinforced polyampholyte gels achieve high toughness under complex loading by combining ionic interactions with secondary reinforcement [7,8,16,17,18,19,20]. Despite this progress, many ionic and polyampholyte gels remain brittle under compression, which limits their use in load-bearing applications [7,21,22,23,24].

Equimolar AMPS–APTAC polyampholytes have been studied in solution and gel form. Prior work examined how charge distribution, salt content, and pH affect swelling, phase behavior, and mechanical properties [23,24,25,26,27]. Other studies introduced hydrogen-bonding motifs into AMPS-based terpolymers or PDMAAm-containing networks and reported improved modulus and toughness when reversible hydrogen bonds act as physical crosslinks [28,29,30,31,32]. These reports show that amide-rich domains can enhance energy dissipation without fully suppressing large deformations [28,29]. However, existing AMPS–APTAC and DMAAm-based systems rarely map compressive properties across a controlled range of monomer concentration, crosslinker content, and DMAAm fraction under identical synthesis and testing conditions [23,24,25,26,27]. A combined analysis of swelling kinetics and compressive failure in such terpolymers is also limited in the current literature [7,21,24].

In this study we address this gap by examining AMPS–APTAC–DMAAm terpolymeric hydrogels with systematically varied initial monomer concentration, MBAAm content, and DMAAm mole fraction. Our working hypothesis is that DMAAm introduces reversible associative interactions that can increase toughness at intermediate composition, while higher monomer and crosslinker contents densify the network and reduce swelling. We further hypothesize that these effects are not monotonic and that an optimal range of DMAAm exists where strength and extensibility are maximized without excessive loss of swelling. To test this, we measure swelling kinetics and equilibrium swelling together with compressive modulus, fracture stress, fracture strain, and toughness across representative compositions.

The novelty and added value of this work lie in four points. First, we provide a systematic composition matrix for equimolar AMPS–APTAC–DMAAm hydrogels that links monomer concentration, covalent crosslinking, and DMAAm content under a single, consistent synthesis protocol. Second, we perform a joint interpretation of swelling and compression data and identify composition-specific deviations from simple crosslink-density trends, including non-monotonic toughness and brittle transitions at high C_x_ and M_γ_. Third, we show that moderate DMAAm levels produce a distinct mechanical regime with high fracture stress and strain, while higher DMAAm contents favor stiffness at the expense of extensibility, in agreement with but not identical to previously reported amide-rich systems. Fourth, we propose a composition–property map that clarifies how AMPS–APTAC–DMAAm networks can be tuned for load-bearing and device-integration scenarios where both swelling and compressive performance are critical.

## 2. Results and Discussion

By adjusting the architectural framework of the network, specifically the crosslinking density and overall monomer concentration, the compressive mechanical properties of polyampholyte hydrogels can be systematically manipulated. These trends are not unique to polyampholyte networks but are commonly observed across diverse hydrogel systems. In this study we describe how these expected behaviors manifest specifically in our AMPS–APTAC–DMAAm terpolymer. An elevation in crosslink density contributes to an increase in the network’s stiffness and enhances the compressive (Young’s) modulus, whereas excessively elevated densities or highly concentrated formulations may compromise the gel’s ductility, rendering it brittle and diminishing its deformative capacity [25,28,29,30]. The Young’s modulus serves as a quantitative metric for assessing stiffness under uniaxial compression conditions and is extensively employed for comparative evaluations of the mechanical properties across diverse hydrogel systems [28,29].

The swelling characteristics are predominantly determined by the ionic chemistry intrinsic to the polymeric network. The type and strength of the ionizable functional groups are crucial in governing pH- and salt-responsive volumetric alterations. Strongly acidic and basic functional groups, such as carboxylate or amino groups, yield more extensive and pronounced pH-dependent swelling behaviors, whereas weaker or less ionizable groups, such as phosphate or sulfonate, reveal limited responsiveness [31,32]. Within polyampholyte networks, the interplay between oppositely charged entities further correlates swelling behavior with ionic strength and pH, resulting in catch-and-release dynamics and adjustable salt sensitivity that are facilitated by electrostatic interactions [32,33]. Therefore, the deliberate optimization of crosslink density, monomer concentration, and charge composition presents a coherent strategy for attaining an optimal equilibrium between toughness and compressive strength in hydrogel systems tailored for load-bearing applications [29,33]. In this work, the term ‘balance’ refers only to the relative improvements observed among our own compositions, because quantitative thresholds for stiffness, strength, and toughness depend on the intended application and are not defined universally.

According to our previous findings, equimolar AMPS–APTAC polyampholyte hydrogels prepared at 5 wt% monomer concentration show improved mechanical properties when crosslinked with 20 mol% MBAAm [24,25]. Increasing the MBAAm concentration raises the crosslinking density and strengthens the network, which improves both mechanical integrity and compressive modulus. When the crosslinking level becomes too high, the network transitions toward excessive rigidity and ultimately becomes brittle [34,35]. This characteristic trade-off is also evident in the present dataset.

**Figure 2:**
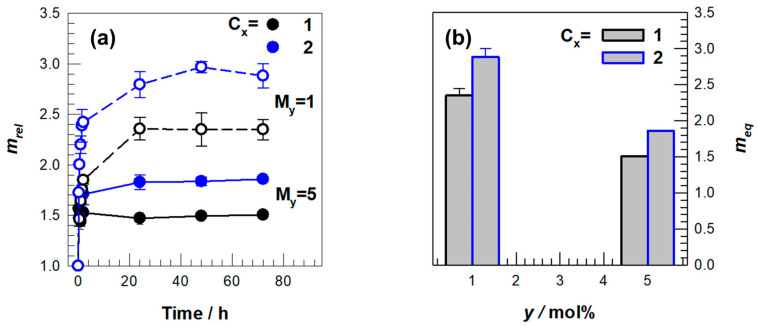
(**a**) Swelling kinetics of the hydrogels prepared different initial monomer C_x_ and chemical crosslinker M_y_ amount. (**b**) Equilibrium swelling degree *m_eq_* of the hydrogels respect to y.

In our study, two separate series of hydrogels were synthesized with initial monomer concentrations (C_x_) ranging from 1.15 M to 1.72 M, MBAAm concentrations (M_γ_) fluctuating between 1 and 5 mol%, and DMAAm contents (D_𝓏_) varying from 8 to 26 mmol to systematically assess mechanical reinforcement and swelling characteristics. The swelling behavior demonstrated a pronounced dependence on the initial monomer concentration (Figure 2). This dependence of swelling on monomer concentration and network density is widely reported for many ionic and non-ionic hydrogels, and our results align with these general principles rather than suggesting polyampholyte-specific behavior. As C_x_ increased, we do not suggest that individual polymer chains became shorter. Instead, the higher monomer concentration increased the effective crosslinking density and produced a denser network with a smaller mesh size, which in turn reduced water uptake and lowered the equilibrium swelling capacity. In contrast, formulations characterized by lower crosslinker content exhibited elevated equilibrium swelling ratios.

The present analysis explicitly interprets how our results align with or diverge from established trends. The plateau in swelling observed for the M_γ_ = 5 samples indicates that crosslinker-driven constraints outweigh charge-mediated swelling at high network densities, and this shift from ionically governed to mechanically restricted behavior is supported by the minimal change in m_rel after approximately twenty hours. The monotonic rise in stiffness with increasing C_x_ is more pronounced than typically reported for lightly crosslinked gels, which suggests that network densification contributes more strongly to mechanical reinforcement than electrostatic repulsion. This pattern likely reflects the dual ionic character of AMPS and APTAC, which promotes tighter chain packing once the network becomes sufficiently concentrated. Overall, the combined swelling and mechanical responses demonstrate that performance is shaped not only by crosslink density but also by the interplay between ionic interactions and chain mobility that is characteristic of the AMPS–APTAC–DMAAm system. These qualitative trends remain consistent with prior findings showing that elevated crosslink density reduces equilibrium swelling and increases stiffness, while excessive crosslinking promotes brittle behavior [34]. De Piano et al. reported strong pH-dependent swelling in polyelectrolyte gels [31], and Zhang et al. described systematic increases in compressive modulus with increasing crosslink density [34]. Modeling studies similarly link monomer composition and crosslink density to network compaction and reduced water uptake in ionically active gels [35,36,37,38,39], which aligns with the behavior observed in our dataset.

**Figure 3:**
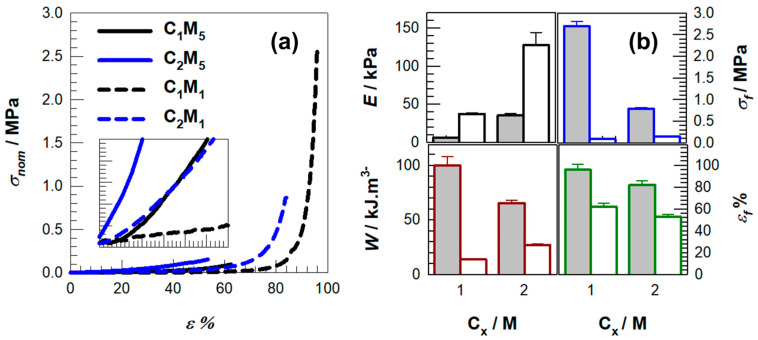
(**a**) Stress–strain curves of hydrogel samples up to around 99% compressions where the nominal stress σ_nom_ is plotted against the compressive strain ε. R = 3 mm.min^−1^. Solid and dashed lines represent the data obtained from MBAAm mol% 1 and 5, respectively. (**b**) Young modulus E, fracture stresses σ_f_, elongation at break ε_f_% and toughness W of the hydrogels plotted against the initial monomer concentration C_x_.

The initial concentration of monomers serves as a critical parameter for influencing the compressive behavior observed in polyampholyte gels. In our investigation, an increase in C_x_ from 1 to 2 M resulted in enhanced resistance to compressive forces and elevated stiffness, which is congruent with the extensive body of literature that correlates increased polymer fraction and effective crosslink density with augmented Young’s modulus and diminished swelling. For instance, an elevation in comonomer concentration at a constant crosslinker level in PNIPAAm systems led to a calculated crosslinking density increase from 297 ± 5 to 405 ± 12 mol m^−3^, concomitantly resulting in a near doubling of compressive strength and an increase in Young’s modulus from approximately 4 to approximately 9 kPa [40] (refer to our trends depicted in Figure 3). Triple-network hydrogels that systematically altered charge content and crosslinker similarly exhibited compressive modulus values nearing approximately 1.8 MPa and strengths around 13 MPa as network density escalated [30].

In addition to mechanical properties, an increase in monomer concentration elevates viscosity and relaxation times within dynamic networks, indicative of denser polymer chain packing and slower rearrangement kinetics [41]. From a microstructural perspective, densification leads to a reduction in effective mesh size and a decrease in interchain distance, which is associated with decreased water absorption and heightened compressive resistance. Contemporary analyses underscore the importance of differentiating between nanoscale mesh and network-level spacing, rather than micron-scale pores, when interpreting the relationship between swelling and mechanical properties [36,42].

While the above trends are expected, Figure 3b reveals several sample-specific features that require closer interpretation. First, the increase in C_x_ does not uniformly enhance all mechanical parameters: the 1.0 → 1.5 M transition produces a disproportionately large rise in Young’s modulus compared to the modest change in fracture stress, suggesting that network stiffening outweighs energy dissipation gains at this composition. Second, the C_1_M_1_ sample exhibits an anomalously high σ_f_ relative to its modest modulus, indicating that low-C_x_ networks preserve chain mobility that delays catastrophic failure despite their lower stiffness. Third, the decline in ε_f_ at the highest C_x_ (2 M) highlights a transition to a more brittle regime, consistent with restricted chain relaxation. These deviations show that the mechanical behavior cannot be explained solely by crosslink-density arguments and instead reflect how ionic pair density and chain mobility co-determine network failure.

Increasing monomer concentration produces a more compact polymer network with shorter effective interchain distances driven by higher crosslinking density, which diminishes equilibrium swelling and enhances compressive strength as shown in Figure 3, although excessive densification can increase the risk of embrittlement [24,25,40,41,42,43,44]. This more compact architecture strengthens the material and reduces its ability to absorb water, a behavior expected for polyampholyte systems that lack intrinsic porosity. The mechanical outcomes summarized in Table 1, including the compressive modulus, toughness, fracture stress, and fracture strain, further illustrate how these structural changes shape performance. A key finding is that toughness does not increase in a simple proportional manner with stiffness or swelling. For instance, the C_1_M_1_ network achieves exceptionally high fracture stress and toughness despite having a relatively low modulus, whereas the C_2_M_5_ network exhibits high stiffness but only moderate toughness. This contrast indicates that although network densification reliably improves stiffness, it does not necessarily produce tougher materials, and optimal performance therefore requires careful balancing of the monomer concentration (C_x_) and crosslinking level (M_γ_).

**Figure 4:**
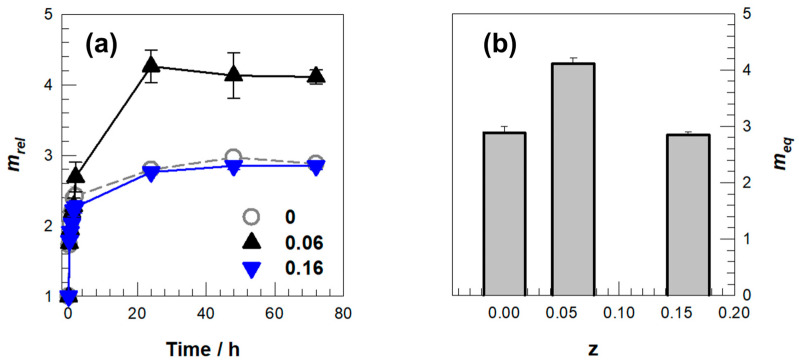
(**a**) Swelling kinetics of hydrogels prepared with (closed) and without DMAAm (open). MBAAm was fixed at 1 mol%. (**b**) Equilibrium swelling degree *m_eq_* of the hydrogels.

The integration of DMAAm into the AMPS–APTAC network introduces additional carbonyl acceptors that participate in reversible hydrogen bonding, thereby augmenting the hydrophilicity of the matrix. Consistent with this role, the samples that incorporate DMAAm exhibit altered swelling kinetics and a distinct equilibrium swelling degree when the concentration of MBAAm is held constant at 1 mol% (Figure 4a,b). Similar reinforcement through hydrogen-bond-assisted mechanisms facilitated by DMAAm segments has been documented in PDMAAm–acid systems, where cooperative donor–acceptor clusters act as physical crosslinks that retain their effectiveness in aqueous environments and across a broad pH spectrum [28,29,30,40]. Quantitatively, compilations from the literature indicate that DMAAm–acid hydrogen-bond networks can enhance stiffness into the MPa range; for example, DMAAm–methacrylic acid systems achieve elastic moduli of approximately 28 MPa at moderate water contents, with similar amide–acid compositions demonstrating a range of approximately 11.5–217 MPa, contingent upon donor identity and network architecture [40].

The enhancement of hydrophilicity attributable to DMAAm is further substantiated by water-structuring measurements: PDMAAm hydrogels exhibit a greater proportion of “intermediate water” in comparison to polyacrylamide, by approximately 12% as indicated in a 2024 study, suggesting an increased affinity for water that may manifest as heightened or expedited uptake dependent on network constraints [30]. Ultimately, the pronounced pH- and salt-responsiveness observed in our terpolymer primarily derives from the oppositely charged AMPS/APTAC pair, whereas DMAAm is expected to influence hydrogen bonding and chain mobility in a manner consistent with previously reported amide-containing networks. Recent investigations into acrylamide-based polyampholyte gels reveal that ionic strength diminishes swelling in polyelectrolytes but exerts minimal influence on electroneutral polyampholytes, while variations in pH significantly affect polyelectrolytes [41], rendering fully charged polyampholytes nearly invariant, trends that elucidate the phenomena observed herein (Figure 4).

The swelling curves in Figure 4 show that DMAAm modulates water uptake through a mechanism distinct from its influence on mechanical strength. The z = 0.06 sample hydrates rapidly and reaches the highest equilibrium mass, indicating that moderate DMAAm incorporation enhances water affinity while preserving sufficient free volume for chains to relax as hydration proceeds. This fast initial uptake suggests that additional hydrophilic sites are introduced without imposing strong constraints on network expansion. In contrast, the z = 0.16 formulation swells more slowly and to a lower final mass, which implies that a higher DMAAm content generates a denser network of hydrogen-bond associations that restrict chain mobility and limit mesh expansion. The reduced early-time swelling and lower plateau value support the interpretation that supramolecular interactions at elevated DMAAm levels act as physical crosslinks that hinder network rearrangement during hydration. These hydration-related constraints parallel the mechanical patterns observed later, because the same associative interactions that increase stiffness in the z = 0.16 network also suppress its swelling capacity, whereas moderate DMAAm at z = 0.06 promotes both water uptake and mechanical dissipation. Consequently, the swelling kinetics reveal how DMAAm simultaneously enhances and constrains network behavior, and they offer an early indication of the performance trade-offs that become more pronounced in the stress–strain response.

**Figure 5:**
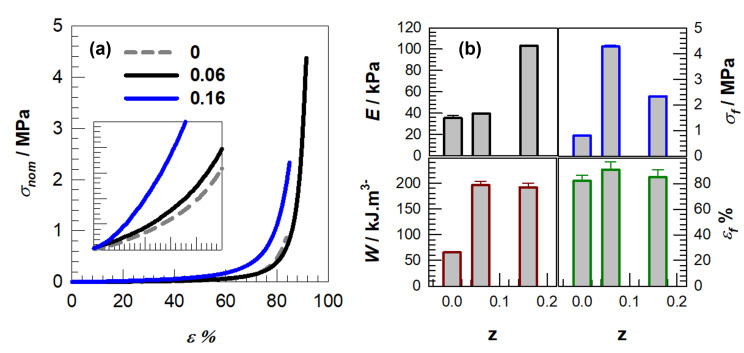
(**a**) Stress–strain curves of hydrogel where the nominal stress σ_nom_ is plotted against the compressive strain ε %. R = 3 mm.min^−1^. (**b**) Young modulus E, fracture stresses σ_f_, elongation at break ε_f_ and toughness W of the hydrogels plotted against the DMAAm mol ratio in the monomer mixture, z.

The stress–strain profiles for z = 0, 0.06, and 0.16 illuminate how DMAAm reconfigures the polymeric network into an architecture that is consistent with hydrogen-bond-stabilized domains, which in turn influence both rigidity and failure characteristics (Figure 5a,b). The integration of a modest fraction of DMAAm (z = 0.06) precipitates an earlier manifestation of strain stiffening and results in the pinnacle of fracture stress, σ_f_ ≈ 4.3 MPa, concomitant with ε_f_ ≈ 91%. An increase in the DMAAm concentration (z = 0.16) amplifies the small-strain stiffness (E) to its apex value within the examined series, while σ_f_ decreases to approximately 2.3 MPa and ε_f_ to roughly 85%, thereby signifying a compromise between stiffness and toughness at heightened association densities.

These behaviors are broadly consistent with supramolecular toughening in amide-rich hydrogels, where carbonyl-based hydrogen bonds act as reversible, load-sharing crosslinks at low strains and as sacrificial, energy-dissipating motifs near failure [45]. Related PDMAAm-based systems preserve cooperative hydrogen-bond clusters across aqueous environments and exhibit enhanced toughness over a wide pH range [46]. Furthermore, prior studies show that modulus typically increases with the number or strength of associative motifs, whereas σ_f_ often exhibits non-monotonic dependences once chain mobility becomes constrained [47,48].

Mechanical testing reveals distinct deformation mechanisms across the three DMAAm compositions and shows that the influence of DMAAm extends beyond simple stiffness enhancement. The z = 0.06 network sustains high stresses while maintaining large fracture strains, which indicates that a moderate amount of DMAAm promotes reversible associations capable of reorganizing under load rather than locking the network at early stages of deformation. The associative domains, which we interpret as hydrogen-bond-rich clusters by analogy with PDMAAm-based systems, appear to distribute stress effectively while preserving chain mobility, which produces the broad strain-stiffening region observed between thirty and fifty percent strain. When the DMAAm fraction increases to z = 0.16, the deformation pathway shifts, as the initial modulus becomes markedly higher while both fracture stress and extensibility decline compared with z = 0.06. This pattern suggests that an abundance of DMAAm-driven amide associations generates rigid domains that cannot relax during large deformation. These domains support small-strain loading but fracture earlier because stress cannot be redistributed efficiently. The mechanical parameters in Figure 5b further confirm this non-monotonic response, since toughness reaches its maximum at z = 0.06 rather than increasing with stiffness, which shows that both strength and ductility depend on dynamic association instead of DMAAm content alone. Overall, the mechanical data indicate that DMAAm adjusts two competing processes, namely load sharing and chain mobility, and it is the balance between these processes that determines whether the network behaves as a tough material or a brittle one.

**Figure 6:**
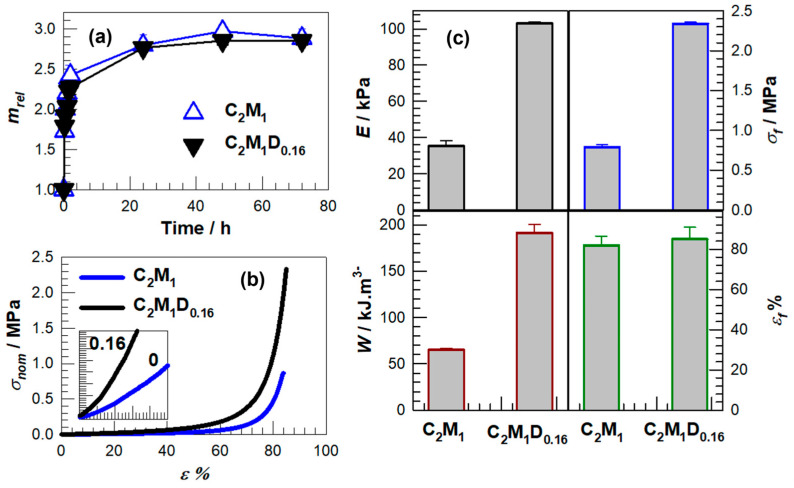
(**a**–**c**) Swelling kinetics (symbols) (**a**), stress–strain curves (lines) (**b**) and mechanical parameters obtained from compression tests (bars) of initial (C_2_M_1_) and improved (C_2_M_1_D_0.16_) hydrogel samples (**c**).

A comparison between the baseline network and the DMAAm-enriched formulation (z = 0.16) highlights how DMAAm simultaneously alters hydration behavior and deformation mechanisms. The z = 0.16 sample reaches a slightly higher equilibrium swelling ratio (Figure 6a), yet its approach to equilibrium is slower, indicating that DMAAm increases water affinity while also generating associative domains that resist rapid network expansion. This dual effect is reflected in the early-time curvature, where the initial uptake is more gradual than in the unmodified system, suggesting that hydrogen-bond clusters limit chain relaxation during the first stages of swelling. The mechanical response mirrors this behavior: the z = 0.16 network displays a steeper small-strain slope and a more extended strain-stiffening region, while the upturn to failure occurs later and at higher stresses (Figure 6b). These features imply that associative interactions, likely involving hydrogen bonding, stabilize the network under low strains and redistribute load during compression without substantially increasing fracture strain. As a result, the increase in toughness observed in Figure 6c arises mainly from energy dissipation through the reversible rupture of associative bonds rather than from enhanced extensibility. Together, the swelling kinetics and mechanical trends demonstrate that higher DMAAm content strengthens the network by promoting load-bearing associative interactions, while simultaneously restricting long-range chain mobility and slowing hydration dynamics.

Overall, these trends reveal a clear composition-dependent trade-off in the z = 0.16 network, where gains in stiffness and work-to-fracture arise alongside penalties in swelling kinetics and extensibility. The increased modulus and toughness suggest that densely populated DMAAm-driven associations efficiently dissipate energy under load, yet the slower hydration and nearly unchanged fracture strain indicate that these same associations restrict chain relaxation and limit large-scale deformation. This interpretation is reinforced by the mechanical data in Figure 6c, where toughness increases sharply despite minimal changes in ε_f_, showing that the material becomes tougher not through enhanced stretchability but through the reversible rupture of associative bonds. Critically, the combined swelling–mechanical analysis demonstrates that the z = 0.16 formulation derives its performance from a balance between enhanced hydrophilicity and constrained mobility, yielding a network that is strong and energy-dissipative but correspondingly slower to swell and less capable of accommodating large deformations.

## 3. Conclusions

Polyampholyte hydrogels have attracted considerable academic scrutiny due to their unique physicochemical characteristics and potential applications across various fields. The swelling and mechanical trends evaluated in this work are not exclusive to polyampholyte networks and are widely reported across many hydrogel systems. A crucial aspect of the investigation of these hydrogels is the clarification of their swelling and compression behaviors, which may provide essential insights into their structural arrangements and mechanical properties. In this analysis, we explored the effects of DMAAm on the swelling and compression responses of AMPS–APTAC hydrogels. The trends observed here are not unique to polyampholyte networks and are widely reported across many hydrogel systems, and our study shows how these general behaviors appear in this specific AMPS–APTAC–DMAAm terpolymer. Empirical investigations have indicated that an increase in DMAAm concentration leads to a modification of the equilibrium swelling ratio of AMPS-APTAC hydrogels. The swelling capability is noted to increase with the addition of a small amount of DMAAm, whereas the swelling capability returns to its original condition upon the application of an excessive amount of hydrogen-bonding DMAAm within the network. In contrast, the incorporation of DMAAm markedly improves the compressive modulus, signifying enhanced stiffness and load-bearing performance. Moreover, the compressive properties of hydrogels are imperative for applications requiring load-bearing functionalities, such as materials utilized in tissue engineering and related fields. The integration of DMAAm into AMPS–APTAC hydrogels has been recognized to significantly augment their compressive modulus. With the rise in DMAAm concentration, the hydrogels exhibit improved stiffness, indicating enhanced mechanical integrity and load-bearing capacity. This phenomenon can be attributed to the reinforcement provided by DMAAm within the hydrogel framework, resulting in increased resistance to compressive forces. Future research in this area may enable the refinement of these hydrogels’ properties for specific applications and enhance their applicability within biomedical and industrial contexts.

## 4. Materials and Methods

### 4.1. Materials

2-Acrylamido-2-methylpropanesulfonic acid sodium salt (AMPS, 50 wt% in water), 3-acrylamidopropyl trimethylammonium chloride (APTAC, 75 wt% in water), and N,N-dimethylacrylamide (DMAAm, 99% purity) were obtained from Sigma–Aldrich (St. Louis, MO, USA). N,N′-methylenebisacrylamide (MBAAm, 99%) was used as the crosslinking agent, and ammonium persulfate (APS, 99%) as the initiator, both purchased from Sigma–Aldrich (St. Louis, MO, USA). All chemicals were used as received without further purification. Deionized water (18.2 MΩ·cm) was obtained using a Milli-Q water purification system (MilliporeSigma, Burlington, MA, USA) and was used in all experiments.

### 4.2. Synthesis of AMPS–APTAC–DMAAm Hydrogels

**Figure 7:**
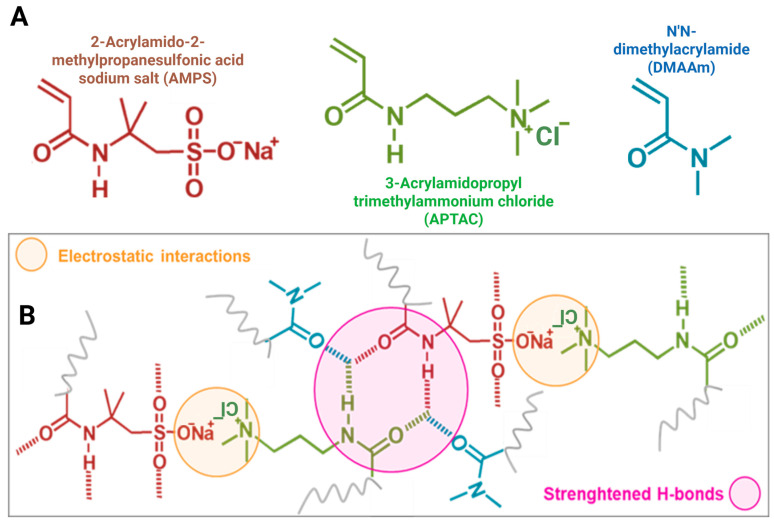
Schematic illustration of the chemical structures and interactions in the AMPS–APTAC–DMAAm terpolymeric hydrogel system: (**A**) Chemical structures of the monomers: AMPS, APTAC, DMAAm; (**B**) Representation of the hydrogel network showing electrostatic interactions between AMPS and APTAC and possible associative interactions involving DMAAm, which are expected to contribute to an effectively higher crosslinking density and mechanical stability.

Building on our previous work on AMPS–APTAC polyampholyte hydrogels [23,24,25,26], new terpolymeric hydrogels were synthesized with the aim of enhancing mechanical performance through additional associative interactions, such as hydrogen bonding suggested in related systems. The ionic balance was maintained by combining equimolar 2-acrylamido-2-methylpropanesulfonic acid sodium salt (AMPS) and 3-acrylamidopropyl trimethylammonium chloride (APTAC) monomers. Polymerization was carried out at 60 °C by free-radical polymerization in aqueous solution using N,N′-methylenebisacrylamide (MBAAm) as the crosslinking agent (1–5 mol% relative to total monomers) and 10 mM ammonium persulfate (APS) as the initiator. The total monomer concentration (C_x_) varied from 1.15 M to 1.72 M. To improve compressive strength, N,N-dimethylacrylamide (DMAAm) was incorporated in mole fractions ranging from 0 to 0.16 (8–26 mmol). The hydrogels were coded as C_x_M_γ_D_𝓏_, where subscripts x, γ, and 𝓏 denote the total monomer concentration, crosslinker content, and DMAAm mole fraction, respectively (Table 2). For instance, C2M1D0.06 corresponds to a total monomer concentration of 1.72 M, 1 mol% MBAAm, and a DMAAm mole fraction of 0.06 (Table 2, Figure 7).

Structural verification of the terpolymer was not repeated in this study because the synthesis procedure follows the same free-radical polymerization protocol that we have previously reported for AMPS–APTAC systems [23,26]. In those publications, successful polymer formation was confirmed by NMR, FTIR, and reproducible gelation behavior. In the present work, gel formation, stable network integrity, and consistent swelling and mechanical responses further support that polymerization proceeded as expected. A note has been added to clarify that structural confirmation relies on our previously validated and published synthesis methodology.

### 4.3. Swelling Tests

Freshly prepared hydrogels were cut into cylindrical pieces (~1 cm length) and immersed in excess deionized water at room temperature until equilibrium was reached. The hydrogels were monitored for 72 h for swelling kinetic measurements. The relative weight (*m_rel_*) swelling ratios of the prepared gels were determined using the following formulas:

(1) $$m_{rel}=m_{swollen}/m_{initial}$$

Here, *m_swollen_* and *m_initial_* represent the mass of the gel specimens in their swollen and after prepared states, respectively.

### 4.4. Mechanical Tests

Uniaxial compression tests were performed at room temperature using a Zwick Roell universal testing machine (ZwickRoell GmbH, Ulm, Germany) equipped with a 500 N load cell. Compression tests were carried out in accordance with ASTM D575. A pre-load of 0.05 N ensured complete contact between the sample and the platens. Tests were carried out at a crosshead speed of 3 mm min^−1^. Stress–strain curves were recorded up to ~99% strain.

Nominal stress ($\sigma_{nom}$) and true stress ($\sigma_{true}$) were calculated using the following relations:

(2) $$\sigma_{true}=\lambda \sigma_{nom}$$

where *λ* is the deformation ratio defined as:

(3) $$\lambda =\frac{l}{l_{0}}$$

The compressive strain (*ε*) was determined assuming constant sample volume, using:

(4) ε=1−λ

## Figures and Tables

**Table 1:** Mechanical properties obtained form compression tests for hydrogels from series. Standart deviations are less than 5% for ε_f_%.

Samples	E/kPa	σ_f/_kPa	ε_f_%	W/kJ.m^−3^
C_1_M_5_	37.3 ± 1.5	90.0 ± 9.5	62.0	13.6 ± 0.6
C_1_M_1_	6.6 ± 0.4	2700.0 ± 110.0	96.0	100.2 ± 7.7
C_2_M_5_	127.3 ± 16.7	150.0 ± 10.0	53.0	27.1 ± 0.7
C_2_M_1_	35.4 ± 2.8	790.0 ± 30.0	82.0	65.6 ± 2.5
C_2_M_1_D_0.06_	39.5 ± 1.0	4280.0 ± 50.0	91.0	196.0 ± 8.0
C_2_M_1_D_0.16_	103.0 ± 0.8	2340.0 ± 20.0	85.0	191.6 ± 9.0

**Table 2:** Experimental conditions for the synthesis of AMPS–APTAC–DMAAm hydrogels (APS fixed at 10 mM).

Effect	Code	Total Ionic Monomers (mol)	DMAAm (mol)	MBAAm (mol)	C_x_ (M)	M_γ_ (mol%)
Concentration	C_1_M_5_	0.0918	—	0.0046	1.15	5
	C_1_M_1_	0.0918	—	0.0092	1.15	1
	C_2_M_5_	0.1376	—	0.0069	1.72	5
	C_2_M_1_	0.1376	—	0.0014	1.72	1
DMAAm	C_2_M_1_D_0.06_	0.1376	0.0083	0.0015	1.72	1
	C_2_M_1_D_0.16_	0.1376	0.0258	0.0016	1.72	1

C_x_ represents the total initial monomer concentration; M_γ_ denotes the molar percentage of crosslinker (MBAAm) relative to total monomers; D_𝓏_ indicates the DMAAm mole fraction in the monomer mixture. APS (10 mM) was used as the initiator in all formulations. Codes correspond to the sample compositions used for comparative evaluation of swelling and compression behavior.

## Data Availability

The original contributions presented in this study are included in the article. Further inquiries can be directed to the corresponding author(s).
